# Investigating the Molecular Genetic Basis of Cytoplasmic Sex Determination Caused by *Wolbachia* Endosymbionts in Terrestrial Isopods

**DOI:** 10.3390/genes9060290

**Published:** 2018-06-08

**Authors:** Myriam Badawi, Bouziane Moumen, Isabelle Giraud, Pierre Grève, Richard Cordaux

**Affiliations:** Laboratoire Ecologie et Biologie des Interactions, Equipe Ecologie Evolution Symbiose, Université de Poitiers, UMR CNRS 7267, Bât. B8, 5 rue Albert Turpin, TSA 51106, 86073 Poitiers CEDEX 9, France; Myriam.Badawi@univ-lemans.fr (M.B.); bouziane.moumen@univ-poitiers.fr (B.M.); isabelle.giraud@univ-poitiers.fr (I.G.); pierre.greve@univ-poitiers.fr (P.G.)

**Keywords:** cytoplasmic sex factor, feminization, sexual development, genome sequencing, gene expression profile, *Wolbachia*, terrestrial isopods

## Abstract

In animals, sexual differences between males and females are usually determined by sex chromosomes. Alternatively, sex may also be determined by vertically transmitted intracellular microbial endosymbionts. The best known cytoplasmic sex manipulative endosymbiont is *Wolbachia* which can, for instance, feminize genetic males into phenotypic females in the terrestrial isopod *Armadillidium vulgare*. However, the molecular genetic basis of cytoplasmic sex determination is unknown. To identify candidate genes of feminization induced by *Wolbachia* strain *w*VulC from *A. vulgare*, we sequenced the genome of *Wolbachia* strain *w*Con from *Cylisticus convexus*, the most closely related known *Wolbachia* strain to *w*VulC that does not induce feminization, and compared it to the *w*VulC genome. Then, we performed gene expression profiling of the 216 resulting *w*VulC candidate genes throughout host developmental stages in *A. vulgare* and the heterologous host *C. convexus*. We identified a set of 35 feminization candidate genes showing differential expression during host sexual development. Interestingly, 27 of the 35 genes are present in the *f* element, which is a piece of a feminizing *Wolbachia* genome horizontally transferred into the nuclear genome of *A. vulgare* and involved in female sex determination. Assuming that the molecular genetic basis of feminization by *Wolbachia* and the *f* element is the same, the 27 genes are candidates for acting as master sex determination genes in *A. vulgare* females carrying the *f* element.

## 1. Introduction

Sex determination is a key biological pathway which governs the sexual differentiation of an individual into a male or female and its ability to produce male or female gametes. Although development as either a male or female is a generally conserved feature in animals, there is an amazing diversity of modes of sex determination [1,2]. The most common mode of sex determination is genotypic sex determination, in which nuclear genetic elements control sex determination. Prime examples of genotypic sex determination are sex chromosomes, i.e., chromosomes that carry sex-determining factors. Sex chromosome systems can take two major forms: male heterogamety (when males are heterozygous for the sex chromosomes and females homozygous, called XY systems), and female heterogamety (when females are heterozygous for the sex chromosomes and males homozygous, called ZW systems). The second mode of sex determination is environmental sex determination, in which external stimuli control sex determination. Environmental sex determination is defined here in a very broad sense, meaning any factor that is not nuclear, be it abiotic or biotic, and can trigger male or female differentiation from a single nuclear genotype. Examples of non-nuclear triggers include temperature, photoperiod, social ties and parasites, among others.

Among parasites, several intracellular microorganisms have evolved the ability to feminize their hosts, a phenomenon known as cytoplasmic sex determination [3,4,5]. Disrupting the mode of sex determination of their hosts in favor of females may be advantageous for such inherited endosymbionts because they are predominantly transmitted vertically through female egg cytoplasm, not male sperm. Thus, males represent dead ends for these microorganisms. Consequently, any effect of the endosymbionts that distort host sex ratio towards females will be selectively advantageous for the endosymbionts, provided that the sex ratio distortion does not unduly affect the ability of hosts to reproduce. The best known causative agent of cytoplasmic sex determination is the α-proteobacterium *Wolbachia*, which has attracted considerable interest, notably for its wide range of hosts and induced phenotypes [6,7]. In particular, several *Wolbachia* strains have been shown to cause feminization of genetic males into phenotypic females [3,4,8]. Feminization has been reported in some insects [9,10] but is most widespread in terrestrial isopod crustaceans, including the common pillbug *Armadillidium vulgare* [11,12,13].

*A. vulgare* has a female heterogametic system of sex chromosomes (ZZ/ZW) [14]. In this context, inherited *Wolbachia* endosymbionts lead to the feminization of ZZ genetic males that develop into fully functional females during sexual differentiation [4,8,11,12]. As *Wolbachia* invades the population, it leads to the disappearance of heterogametic females (ZW) and therefore, to W sex chromosome extinction. Effectively, this means that the female sex-determining factor shifts from nuclear (W sex chromosome) to cytoplasmic (*Wolbachia*) localization [15]. Interestingly, *Wolbachia* horizontally transferred its genome into the host nucleus, in a genomic region associated with female sex-determination called the *f* element [16]. The *f* element ultimately led to the birth of a new W sex chromosome [16]. Thus, *Wolbachia* is not only a factor inducing cytoplasmic sex determination, it also has the potential to act as a substantial evolutionary force that can trigger turnovers of sex chromosomes in *A. vulgare*. However, the mechanisms and genes responsible for feminization by *Wolbachia* and the *f* element are unknown [15].

In general, the molecular mechanisms underlying *Wolbachia*-host interactions remain poorly known (reviewed in [17]). This is largely due to the fact that *Wolbachia* is an unculturable bacterium with an obligate intracellular lifestyle. With respect to feminization, genome sequencing of the feminizing *Wolbachia* strain *w*VulC identified many genes that could potentially interact with its host *A. vulgare*, especially genes that encode for proteins containing eukaryotic-like domains (at least 83 putative genes including 75 with ankyrin domain; Figure S1). These domains are commonly found in eukaryotic proteins and are relatively rare in bacteria, but particularly abundant in *Wolbachia* genomes [18]. Genes containing eukaryotic-like domains are particularly enriched in prophage WO, which is present in many *Wolbachia* genomes, sometimes in multiple copies [19,20,21]. These genes are of particular interest as their role in host-pathogen interactions has been demonstrated in other microorganisms such as *Legionella pneumophila* [22,23], *Coxiella burnetii* [22,24] and *Anaplasma phagocytophilum* [25]. In *Wolbachia*, it has already been shown that some of these genes show sex-specific expression [26] or are correlated to the induced phenotype [27,28,29]. In several *Wolbachia* strains that infect terrestrial isopods, the ankyrin gene *pk2b2* (embedded in one of the 7 prophage regions of the *w*VulC genome; Figure S1) is only expressed in females from species infected by feminizing strains [30]. Sequencing of the *w*VulC genome also revealed the presence of numerous export and secretion systems such as T4SS, T1SS, Sec, Tat, Lol and Bam, through which effectors may transit towards host cytoplasm. This result is consistent with earlier reports, indicating that the presence of such systems is a general feature of *Wolbachia* endosymbionts [31,32,33]. In *w*VulC, the T4SS has been well studied [31,34]; however, no effector or molecular mechanism of feminization induced by *Wolbachia* has been uncovered to date. This is in contrast with cytoplasmic incompatibility (CI), the most common phenotype induced by *Wolbachia*, which has recently been shown to involve the pair of genes, *cifA* and *cifB* [35,36,37].

So far, many studies aiming at identifying candidate genes involved in *Wolbachia*-host interactions used targeted approaches, i.e., by focusing on genes containing functional domains with prior knowledge of involvement in molecular interactions. Considering the quite limited amount of functional knowledge of *Wolbachia* genes (e.g., 23% of hypothetical genes in *w*VulC genome), it may be relevant to consider alternative strategies to identify candidate effectors to elucidate molecular mechanisms of induced phenotypes [38]. In this study, we used a combination of comparative genomics and expression profiles throughout host development to identify candidate genes of feminization induced by *Wolbachia w*VulC, without prior functional knowledge of genes. Specifically, we sequenced the genome of *Wolbachia w*Con from the terrestrial isopod *Cylisticus convexus* and compared it to the *w*VulC genome. *w*Con was selected because it is the most closely related known strain to *w*VulC available in our laboratory that does not induce feminization (but CI) [39,40]. In addition, it has been shown that *w*VulC also induces feminization when transfected into *C. convexus* and *w*Con also induces CI when transfected into *A. vulgare* [39,41], indicating that the observed phenotypes are related to the *Wolbachia* strains. Next, we performed gene expression profiling of selected *w*VulC genes throughout host developmental stages in *w*VulC native host *A. vulgare* and following *w*VulC transfection in a heterologous host (*C. convexus*). *C. convexus* was selected for comparison with *A. vulgare* because: (i) *w*VulC has been shown to induce feminization after transfection in *C. convexus*, and (ii) *A. vulgare* and *C. convexus* differ in their timing of sexual differentiation [41]. Using this strategy, we identified a set of 35 candidate genes out of the 1888 *w*VulC genes that may be implicated in feminization induced by *Wolbachia w*VulC in the host *A. vulgare*, two of which appear to be particularly promising.

## 2. Materials and Methods

### 2.1. Terrestrial Isopod Lines

All *C. convexus* individuals naturally infected with *Wolbachia* strain *w*Con were from our laboratory line CCw, derived from individuals caught in Avanton, France, in 2004. All *C. convexus* individuals carrying *Wolbachia* strain *w*VulC were from our laboratory line AW (derived from individuals caught in Villedaigne, France, in 1997) and were experimentally infected with *Wolbachia* strain *w*VulC (from our laboratory line ZN, derived from individuals caught in Celles sur Belle, France, 1991), as described in Badawi et al. [41]. All *A. vulgare* individuals naturally infected with *Wolbachia* strain *w*VulC were from our laboratory line ZN. Isopods were reared at 20 °C with food ad libitum (dead lime tree leaves and carrots) under a natural photoperiod, except those in cross-breeding and juveniles which were reared under a 18L:6D photoperiod.

### 2.2. wCon DNA Enrichment and Genome Sequencing

To identify the *C. convexus* tissue naturally possessing the highest *Wolbachia w*Con density, DNA was extracted from ovaries, the nervous chain and the hemolymph of four *C. convexus* females using a standard phenol/chloroform extraction [42]. Proportions of *w*Con, mitochondrial and nuclear DNA were estimated with quantitative PCR targeting single copy genes *wsp*, cytochrome oxidase I (COI) and androgenic hormone (AH), respectively. Reactions and cycle conditions were as described in Le Clec’h et al. [43] and in Table S1. Copy numbers were estimated using calibration range curves as described in Le Clec’h et al. [43]. We assessed the ratios of the base pair number from mitochondria (R_mt_) and nucleus (R_nuc_) relative to the *w*Con genome, as follows: R_mt_ = (G_mt_ × N_COI_)/(G_Wo_ × N_wsp_) and R_nuc_ = (G_nuc_ × N_AH_)/(G_Wo_ × N_wsp_), where G_mt_, G_nuc_ and G_Wo_ are mitochondrial, nuclear and *Wolbachia* genome sizes, respectively, and N_COI_, N_AH_ and N_wsp_ are COI, AH and *wsp* copy numbers, respectively. Absolute genome sizes were estimated as 1.7 Mb and 2 Gb for *w*Con and nuclear genomes respectively, based on information available for the closely related *Wolbachia w*VulC/*A. vulgare* complex (http://www.genomesize.com/). *C. convexus* mitochondrial genome length is 14 kb, based on sequencing data [44].

Ovaries were selected for further analyses as they contained the highest ratio of *w*Con copies. We dissected ovaries, as well as nerve cords of 30 *C. convexus* sisters infected with the *w*Con *Wolbachia* strain. First, we performed PCR amplification and Sanger sequencing of the *Wolbachia*-specific markers *wsp* and *ftsZ* using the DNA of the nerve cords from each of the 30 females as templates and then verified that the *Wolbachia* strain was indeed *w*Con (see Table S1 for primers and PCR conditions). Next, to enrich the sample in *w*Con DNA, we homogenized the ovaries of the 30 females with a Dounce tissue grinder B within a PBS solution containing sucrose (0.25 M) and l-glutamine (5 mM) to crush cells while keeping nuclei intact. We then passed the solution through a 5 µm filter that specifically retained nuclei but not *Wolbachia*. Next, the DNA of the *Wolbachia*-enriched solution was extracted using a standard phenol/chloroform procedure. RNA contaminants were removed with RNase A treatment (0.2 µg/µL at 37 °C for 1 h). Then, the sample was purified through another round of phenol/chloroform extraction. We determined the relative proportion of *w*Con DNA relative to mitochondrial and nuclear DNA with quantitative PCR (qPCR), as described above. The DNA sample was used by GenoScreen (Lille, France) to prepare a Nextera sequencing library. The library was sequenced by GenoScreen in a 1/4 and a 1/8 454 GS FLX sequencer runs with Titanium chemistry.

Bad quality reads and mitochondrial reads were filtered out and the remaining reads were assembled de novo with gsAssembler (Newbler 2.6) with default parameters except seed step set to 1, yielding 5153 contigs. *w*Con contigs were retrieved using BLAT (minimal identity set to 60), Mauve and R2CAT [45,46,47]. Six representative *Wolbachia* reference genomes from supergroups A and B were used to retrieve *w*Con contigs: *w*Mel (NC_002978.6), *w*Ri (NC_012416.1), *w*Pip-Pel (NC_010981.1), *w*AlB (NZ_CAGB00000000.1), *w*VitB (NZ_AERW00000000.1) and *w*VulC (ALWU00000000). The assembly of 389 contigs showing high similarity with *Wolbachia* genomes were manually improved by exploiting the de Bruijn graph to join contigs truncated due to repeats, which resulted in a final *w*Con assembly of 237 contigs. Gene prediction and annotation was performed with PROKKA pipeline [48] and manually curated in artemis. Clusters of Orthologous Groups of proteins (COG) were assigned to each predicted protein using BLASTp with minimal identity of 70% on at least 70% of the protein sequences. The annotated genome sequence and raw sequence data were deposited in GenBank under bioproject accession number PRJNA439208.

### 2.3. In Silico Classification of wVulC Genes

To determine a list of feminization candidate genes, all annotated genes from the *w*VulC genome (GenBank accession number ALWU00000000) were processed using a homemade pipeline of Perl scripts (Supplementary Materials 1). First, according to genome annotation, all repeats (including transposable elements, genes coding for prophages and other repeated genes), pseudogenes (including all coding sequences of genes annotated as “pseudogenes” and small coding sequences of genes split in two chunks) and small peptides (<50 amino acids) were removed. Repeats were discarded because they were not well suited for downstream expression analyses by PCR. Pseudogenes and small peptides were discarded as they are unlikely to be functional genes. Second, all genes belonging to the core genome of *Wolbachia* strains that infect arthropods were discarded. This core genome was computed using OrthoMCL [49] with a minimal identity of 70% and a minimal e-value of 10^−6^, using the following five complete *Wolbachia* genomes from supergroups A and B: *w*Mel (NC_002978.6), *w*Ri (NC_012416.1), *w*Ha (NC_021089.1), *w*No (NC_021084.1), *w*Pip-Pel (NC_010981.1). Finally, all proteins from *w*VulC showing 100% similarity with their homolog in the closely related, non-feminizing *w*Con genome on at least 90% of their length, based on BLASTp searches, were discarded. The remaining genes were considered as candidates and were selected for expression analyses.

### 2.4. Expression of wVulC Candidate Genes throughout Host Sexual Development

To investigate the expression of *Wolbachia* candidate genes during host sexual development, genetic crosses involving *A. vulgare* and *C. convexus* individuals were performed between uninfected males and females carrying the *w*VulC *Wolbachia* strain. In the terrestrial isopods *A. vulgare* and *C. convexus*, embryos that inherited the feminizing *Wolbachia* strain *w*VulC develop into functional females. This conversion of genetic males into functional phenotypic females occurs during host sexual differentiation. In brief, *A. vulgare* juvenile development occurs within a period of 10 to 15 weeks after the release of juveniles from the female ventral pouch. During this period, eight post-embryonic stages were defined, each corresponding to an intermolt stage. Gonads differentiate during stages 4 to 6 and after this period the experimental reversion of gonadal sex becomes impossible [50,51,52]. Therefore, *Wolbachia* is assumed to act before or during sexual differentiation as it inhibits male gonad differentiation and hence converts genetic males into phenotypic females. *C. convexus* has a different sexual differentiation timing compared to *A. vulgare*, as it shows a one-stage shift and occurs during stages 3 to 5 [41]. After hatching, juveniles were sampled individually 2–3 days after each molt, from the first molt (stage 1) to the seventh molt (stage 7), and stored in liquid nitrogen.

First, we tested whether *w*VulC candidate genes are expressed in any of the gonadal differentiation key stages (3 to 6) in *A. vulgare* juveniles. We co-extracted DNA and RNA of 3, 2, 2 and 2 juvenile pools sampled from stages 3 to 6 respectively, using the AllPrep DNA/RNA kit (Qiagen^®^, Venlo, The Netherlands), according to the manufacturer’s instructions. We confirmed *w*VulC presence in each sample by amplifying molecular markers *gatB* and *wsp* by PCR (Table S1 for primers and PCR conditions). RNA samples were then treated with DNase I (4u at 37 °C for 1h and inactivated at 75 °C for 10 min, New England Biolabs^®^, Ipswich, MA, USA) and cDNA synthesis was performed using the Superscript SSIII kit (Invitrogen^®^, Carlsbad, CA, USA), according to the manufacturer’s instructions, and using 250 ng of RNA (estimated with Nanodrop, Thermo Fisher, Waltham, MA, USA). Then, RT-PCR was performed on all candidate genes (Table S2 for primers and PCR conditions) and PCR products were run on an agarose gel 1.5%. After staining the gel for 10 min in an ethidium bromide bath, bands were revealed under UV light. Candidate genes being expressed during at least one host developmental stage were selected for expression quantification.

*Wolbachia w*VulC gene expression was quantified by quantitative real-time PCR (qRT-PCR) on three successive samplings. The first sampling was composed of 40, 24, 16, 16 and 16 *A. vulgare* juvenile pools sampled from stages 2 to 6, respectively. The second sampling was composed of triplicated pools of 10, 10, 9, 6, 6, 6 and 6 *A. vulgare* juveniles corresponding to stages 1 to 7, respectively. The third sampling was composed of triplicated pools of eight *C. convexus* juveniles for each stage from 1 to 7. All quantitative experiments were conducted with two technical replicates, using a LightCycler LC480 device (Roche^®^, Basel, Switzerland). Reactions were made in a total volume of 10 µL composed of 1× Fast SYBR GREEN Master Mix (Roche^®^, Basel, Switzerland), 5 µM of each primer, and 2.5 µL of 4×-diluted cDNA synthesized as above. Cycle conditions were as described in Le Clec’h et al. [43].

To identify appropriate reference genes, we measured the expression of 15 *Wolbachia* housekeeping genes (*wsp*, *coxA*, *atpD*, *sucB*, *gatB*, *gltA*, *L2*, *L20*, *S4*, *fabF*, *pyrB*, *purF*, *tkt*, *hcpA* and *fbpA*) by qRT-PCR in *A. vulgare* stages 2 to 6. We then selected those exhibiting a highly correlated expression (*r* > 0.95) using the BestKeeper software (version 1) [53] (Table S1 for primers and PCR conditions). At last, we verified that the expression of the selected genes remained constant across stages in *A. vulgare* and *C. convexus* relative to the *wsp* copy number. This procedure resulted in the validation of six genes: *gltA*, *L2*, *L20*, *S4*, *fabF* and *purF*. Expression ratios were calculated by combining efficiency and 2^ΔΔct^, according to LightCycler 480 SW v1.5 software manual (Roche^®^, Basel, Switzerland). Efficiency and cycle threshold were assessed by real-time PCR (RT-PCR) Miner [54]. Then, each expression ratio was calibrated against stage 2 (corresponding to the earliest stage for which all genes were amplified in all samplings). Under- and over-expression were considered with thresholds of 0.5 and 2, respectively for all successive samplings.

To compare *w*VulC gene expression patterns between *A. vulgare* and *C. convexus* hosts, we used cross-correlation tests using R software [55]. This test measures correlation by taking into account gene expression patterns shifted between stages (hence taking into account the one-stage shift of sexual differentiation that occurs between the two hosts [41]). This test was applied after an autocorrelation test aimed at verifying the absence of autocorrelation between the different time steps. In positive cross-correlation tests, we computed the η^2^ value to verify that the variance did not influence the cross-correlation test.

### 2.5. Functional Annotation of Candidate Genes

Function prediction of the final set of candidate genes was performed by searching the following databases: PFAM [56], SMART [57], cdd [58], cog [59], kog [60] and signal peptide [61]. Results were accounted as significant with an e-value <10^−4^.

### 2.6. Identification of Candidate Genes in the f Element

Considering the *f* element as the *Wolbachia* nuclear insert characterized by Leclercq et al. [16], the presence of the final set of feminization candidate genes from *w*VulC in the *f* element was investigated using BLASTp against *f* element sequences sensu Leclercq et al. [16] (GenBank accession numbers LYUU01002088.1-LYUU01002096.1), with a maximal e-value set to 0.001 and at least 90% of identity.

## 3. Results

### 3.1. Sequencing of wCon Wolbachia Genome

**Figure 1:**
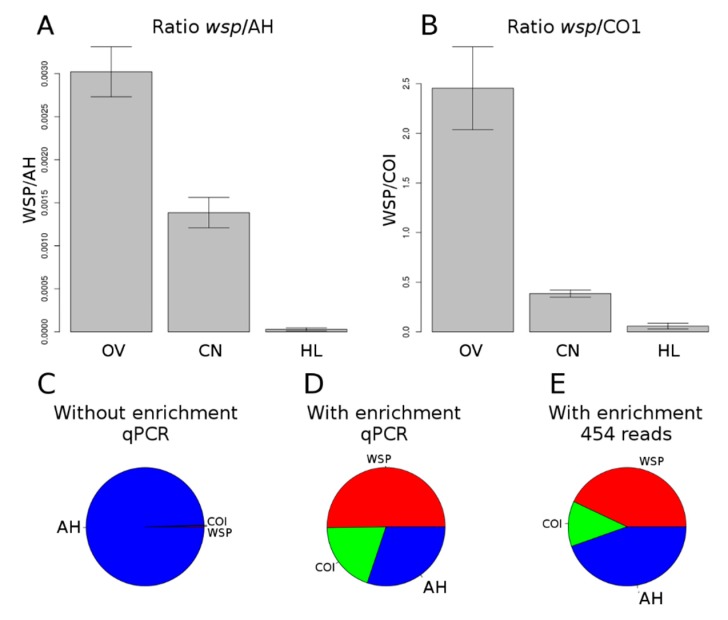
Ratios of *wsp* gene copy number relative to androgenic hormone (AH) copy number (**A**) and cytochrome oxidase I (COI) copy number (**B**), in ovaries (OV), nervous cord (CN) and hemolymph (HL). Proportions of base pairs based of *w*Con (red), mitochondrial (green) and nuclear genomes (blue) estimated by quantitative PCR (qPCR) without (**C**) and with (**D**) enrichment, and by mapping of pyrosequencing reads on assembled genomes (**E**). WSP: please define.

To sequence the *w*Con *Wolbachia* genome, we first applied an enrichment method, as *Wolbachia* DNA is often in low abundance compared to host genetic material (nuclear and mitochondrial). To evaluate which *C. convexus* tissue contains the highest abundance in *Wolbachia* DNA, we quantified three single-copy genes by means of qPCR (androgenic hormone for nuclear DNA, cytochrome oxidase I for mitochondrial DNA and *wsp* for *Wolbachia* DNA) in three different tissues (gonads, hemolymph and nervous cord). The highest level of *w*Con DNA was found in gonads (~1 *Wolbachia* bp for ~330 nuclear bp), followed by the nervous cord (~1 *Wolbachia* bp for ~720 nuclear bp) and hemolymph (~1 *Wolbachia* bp for ~34,720 nuclear bp) (Figure 1A). These figures correspond to 1–4 *Wolbachia* cells per host cell on average. The quantity of mitochondrial nucleotides was lower than that of *Wolbachia* (~1 *Wolbachia* bp for ~0.4 mitochondrial bp in ovaries; Figure 1B). Subsequently, we applied our DNA extraction and enrichment protocol to a pool of 30 gonad pairs from *C. convexus* sisters, reaching a >300-fold enrichment in *w*Con DNA (Figure 1C,D).

Sequencing of this sample generated 395,850 reads representing a total of 110,934,433 bp. 16,491 reads were filtered out for low quality and 47,065 reads that mapped against *C. convexus* mitochondrial genome were discarded. In total, 163,099 reads (43%) were mapped against the final set of *w*Con contigs. Therefore, qPCR estimation of *w*Con DNA proportion (50%) was close to that obtained by sequencing (Figure 1D,E). From these reads, we assembled the *w*Con *Wolbachia* genome in 237 contigs, with a total size of 2.1 Mb, an average sequencing depth of 25×, a N50 of 4.5 kb and a GC content of 34.7%. Contig size ranged from 404 to 71,427 bp. PROKKA annotation pipeline revealed the presence of 2359 coding sequences with an average size of 702 bp per gene, 35 transfer RNA (tRNA) and 3 ribosomal RNA (rRNA) genes (Table 1). Genome size is likely overestimated since redundant repeats are present at the ends of contigs resulting from manual scaffolding, as described in Section 2.2. Consistently, assignment of annotated coding DNA sequences (CDS) to COG categories revealed that *w*Con contains a high number of genes in the category “Replication, Recombination and DNA Repair” among the 7 *Wolbachia* genomes compared (Figure S2). This COG category includes transposable elements (402 repeats in *w*Con).

Core genome analysis performed on five complete *Wolbachia* genomes from arthropods (*w*Mel, *w*Pip-Pel, *w*Ri, *w*Ha and *w*No) revealed the presence of 762 orthologous groups in their core genome. We found 752 and 755 of these orthologous groups in the *w*Con and *w*VulC genomes, respectively (748 of which are shared by *w*Con and *w*VulC). Furthermore, 35 tRNA and 3 rRNA genes were characterized in both *w*Con and *w*VulC genomes (Table 1). Altogether, these analyses indicate that we obtained reliable assemblies of the *w*Con and *w*VulC genomes, capturing most of the genetic information despite assembly fragmentation (237 and 10 contigs for *w*Con and *w*VulC, respectively).

### 3.2. In Silico Determination of Candidate Genes

The *w*VulC *Wolbachia* genome contains 1888 annotated coding sequences. A succession of in silico filters (see Materials and Methods) allowed us to establish a set of feminization candidate genes. As a result, based on the *w*VulC annotation, we excluded all repeats (*n* = 792), pseudogenes (*n* = 26), genes <150 bp (*n* = 52) and small CDS of genes split in two CDS (*n* = 16). We also removed genes orthologous to the ones identified in the core genome of five *Wolbachia* strains of arthropods (*w*Mel, *w*Ri, *w*Pip-Pel, *w*Ha and *w*No) (*n* = 721). This number is different from the above core genome analysis (*n* = 755) because a subset of core genes was discarded as part of previous filters. Next, remaining *w*VulC proteins were compared to their homologs in the non-feminizing *w*Con *Wolbachia* genome (CI-inducing strain), and those showing 100% similarity (*n* = 16) were excluded (Table S3, Figure S1). The 265 remaining *w*VulC genes were considered as a first set of feminization candidate genes and were processed for expression studies.

### 3.3. Candidate Gene Expression throughout Host Development

Among the 265 remaining *w*VulC genes, successful PCR amplification was obtained for 216 candidate genes. Then, gene expression of the 216 candidate genes from the *w*VulC *Wolbachia* genome was investigated by RT-PCR throughout *A. vulgare* development, in particular during the stages of sexual differentiation, i.e., from stages 3 to 6. We found that 139 out of 216 *w*VulC genes were expressed during at least at one of these host developmental stages (Table S3). Next, gene expression of the 139 candidate genes was investigated by qRT-PCR throughout developmental stages of the host *A. vulgare*, using two successive samplings. First, we investigated expression profiles of these genes during host developmental stages 2 to 6, using stage 2 for calibration (see Materials and Methods, Figure S3). This analysis identified 13 and 29 genes that were 2-fold under and over-expressed, respectively, in at least one of stages 3 to 6. Expression profiles of these 42 differentially expressed genes were then investigated within the frame of a second sampling. The second sampling was designed to confirm and refine expression profiles of *w*VulC candidate genes throughout the full development sequence of the host *A. vulgare* (i.e., from stages 1 to 8)*.* In total, seven genes were below the 2-fold expression threshold for all replicates. Therefore, 35 genes were consistently under or over-expressed, making them the final set of candidate genes. 

**Figure 2:**
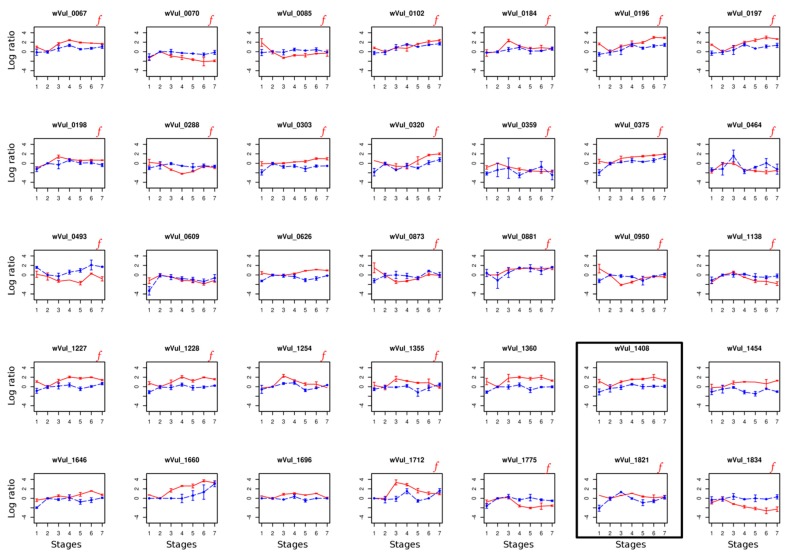
Candidate gene log2 expression of 35 candidate genes during both homologous and heterologous host development (*A. vulgare* in red and *C. convexus* in blue, respectively). All candidate genes are consistently over- or under-expressed during native host development (in *A. vulgare*). All genes present in the *f* element are labeled with a red “*f*”. The two genes showing a significant cross-correlation with exactly one-stage shift between the two hosts are boxed.

Finally, expression profiles of the 35 *w*VulC candidate genes were investigated, during developmental stages 1 to 7 in the heterologous host *C. convexus*, in which *w*VulC transfection was shown to be associated with feminization (Figure 2) [41]. We found that all 35 *w*VulC genes were expressed during *C. convexus* development, but only 29 of these genes were significantly down or up-regulated in both hosts (Figure 2, Table S3). A cross-correlation analysis of gene expression profiles between the two hosts indicated that three *w*VulC genes exhibited the same gene expression profile throughout the entire developmental sequence in both hosts: wVul_1408 and wVul_1821 with one-stage shift that perfectly matches the one-stage shift of sexual differentiation existing between *A. vulgare* and *C. convexus* (Figure 2) [41], and wVul_0881, but without any stage shift between the two hosts (Figure S4).

### 3.4. Prediction of General Function of wVulC Candidate Genes

We investigated the general function of the 35 *w*VulC candidate genes that are consistently over or under-expressed throughout *A. vulgare* development. We were able to infer functions for 12 of these genes, in which we found a total of 14 different domains (up to 4 domains per gene) (Table 2; Table S4). We unraveled the following predicted domains associated with:

1. Eukaryotic-like domains involved in protein-protein interactions: ANK (Ankyrin Repeat) [62,63] in wVul_0303, wVul_1408 and wVul_1775; LRR (Leucine Rich Repeat) [64,65] in wVul_0493; TPR (Tetratricopeptide Repeat) [66] in wVul_0881;
2. Cytoskeleton: Hook in wVul_0067 that mediates attachment to microtubules [67]; Spc7 in wVul_0375 that is required for kinetochore-spindle association during cell division [68];
3. DNA binding: Smc (Structural Maintenance of Chromosomes) in wVul_0067, wVul_0085 and wVul_0375 that binds DNA and acts in organization and segregation of chromosomes for partition [69,70]; AAA (ATPases Associated with diverse cellular Activities) [71] in wVul_0375 that are involved in diverse cellular functions including DNA replication, gene expression and also vesicle-mediated transport and peroxisome assembly; p53 interaction protein in wVul_1775 that is responsible for DNA binding [72].
4. Hydrolase function: Lipase class 3 in wVul_0873 that hydrolyses ester linkages of triglycerides [73,74]; Peptidases/proteases that hydrolysepeptidic bounds [75,76] in wVul_1408, wVul_1454 and wVul_1775;
5. Membranes: Mitofilin in wVul_0085 that is an inner membrane protein domain of mitochondria that controls cristae morphology [77,78]; IncA in wVul_1360 that is associated with homotypic fusion of inclusions in the obligate intracellular bacterium *Chlamydia trachomatis* [79]; OmpA-like superfamily in wVul_0359 that is an outer membran protein domain [80]; Trp in wVul_1408 and wVul_1775 that is a transient-receptor-potential calcium channel protein [81].

### 3.5. Presence of Candidate Genes in the f Element

We searched the *f* element sequence for the 35 *w*VulC candidate genes. Blast results indicated that 27 genes are present in the *f* element with copy numbers varying from 1 to 5 (Table S5). The 8 *w*VulC candidate genes with no hit in the *f* element were wVul_0609, wVul_0626, wVul_1360, wVul_1408, wVul_1454, wVul_1646, wVul_1660 and wVul_1696. Among the 27 genes present in both *w*VulC and the *f* element, 20 presented identical copies at the amino acid level (Table S5). The lowest amino acid identity was observed for wVul_0198 (95.35% identity). There was no significant enrichment of differentially expressed genes in the *f* element (Fisher’s Exact test, *p* = 0.22).

## 4. Discussion

Our analyses resulted in the identification of a reduced set of 35 candidate genes that may be implicated in *Wolbachia w*VulC-mediated feminization in the native host *A. vulgare* and the heterologous host *C. convexus*. The vast majority of *w*VulC genes that we tested are consistently expressed throughout the entire host development sequence, as previously observed for *Wolbachia w*Mel in its host *Drosophila melanogaster* [82]. Only one-fourth of genes analyzed by qRT-PCR (35/139) are expressed differentially, of which only one-third are functionally annotated (12/35). For example, we noted the presence of several eukaryotic-like domain proteins (ANK, TPR and LRR) and an outer membrane protein (OmpA-like) in our set of candidate genes. Identifying genes encoding such domains among our candidates is not really surprising, as previous host/*Wolbachia* molecular interaction studies have often searched for such functional domains when using targeted strategies to identify candidate genes [27,28,29]. One advantage of the strategy we used here was the ability to identify candidate genes without any a priori knowledge of their function. This allowed us to identify functionally annotated candidate genes among the “usual suspects”, as well as candidate genes for which no functional information is available. Genes from the latter category may be prime choices for future functional studies, in the context of feminization induced by *w*VulC. This point may be relevant in identifying *Wolbachia* effectors, as exemplified by *cifA* and *cifB,* and their homologs *cidA* and *cidB*, which were not functionally annotated before their identification as CI-effectors in *w*Mel and *w*Pip-Pel strains, respectively [35,36].

The candidate gene approach we used also has its own potential limitations. First, as it largely relies on gene expression patterns, we implicitly assumed that gene expression and protein expression are correlated, which may not necessarily be true. This assumption is, however, generally made by studies involving gene expression. Second, we opted to discard core genes and mobile genetic elements. In particular, the rationale for removing mobile genetic elements was their repeated nature that makes them poorly suited to the design of specific PCR assays. Nevertheless, it is clear that genes encoded by mobile genetic elements may be involved in host-*Wolbachia* interactions, as perfectly exemplified by the *cifA* and *cifB* genes that are located in prophage WO [35,36]. However, our strategy did not dismiss those genes as candidate genes. Instead, we opted to give them lower priority in our expression analyses. Third, our approach is based on gene expression profiling throughout host development. However, during host development, *Wolbachia* is likely involved in various biological processes unrelated to feminization. For example, while host cells are dividing, *Wolbachia* cell population is also in expansion, replicating fast and colonizing new cells [83,84,85,86,87,88]. Therefore, a subset of our candidate genes may have passed our successive filters not because they are involved in feminization but because they are involved in a confounding biological process taking place during host sexual development. 

In this context, the three domains Smc, Hook and Spc7 found in the wVul_0067, wVul_0085 and wVul_0375 candidate genes, are consistent with the involvement of these candidate genes in cell division [67,68,69,70]. Moreover, *Wolbachia* cell multiplication requires lipid recruitment to generate new membranes and additional nutrient supply. Thus, it is conceivable that the wVul_1360 candidate gene containing an IncA domain may contribute to lipid recruitment, as it seems to act in *C. trachomatis* [79,89,90]. This may also explain the presence of several lipase or peptidase domains among our candidate genes, which may contribute to additional nutrient supply. Interestingly, two candidate genes (wVul_1408 and wVul_1775) possess both a eukaryotic-like domain (ANK) and a peptidase/protease domain, suggesting that *Wolbachia* might cleave host peptides.

Among the 35 feminization candidate genes we identified, 27 were also found in the *f* element, which is a piece of a feminizing *Wolbachia* genome horizontally transferred in the nuclear genome of *A. vulgare* and involved in female sex determination [16]. Assuming that the molecular genetic basis of feminization by *Wolbachia* and the *f* element is the same, the 27 aforementioned genes are candidates for acting as master sex determination genes in the *A. vulgare* genome. However, in spite of the high nucleotide similarity of these genes in *w*VulC and the *f* element, their function or regulation may not necessarily be conserved in the two genetic entities. This may be the case especially when considering that *Wolbachia* genes are located in the cytoplasm and controlled in a prokaryotic context while *f* element genes are located in the nucleus and controlled in a eukaryotic context. Furthermore, feminization induced by *Wolbachia* and the *f* element presents differences (reviewed in [15]). For example, it is possible to experimentally masculinize young females carrying the *f* element by grafting of androgenic glands [91]. By contrast, young females infected by *Wolbachia* are not masculinized using the same experimental procedure [92,93]. Thus, the underlying mechanisms of feminization by the *f* element and *Wolbachia* may differ, at least partly. Nevertheless, given that the *f* element is derived from *Wolbachia*, it is parsimonious to consider that the molecular genetic basis for feminization shows at least some overlap between the two feminizers. For this reason, it is relevant to consider the 27 *Wolbachia* feminization candidate genes present in the *f* element as candidate genes for master sex determination in females carrying the *f* element. In this context, it will be useful to investigate the expression profiles of these candidate genes during sexual development of *A. vulgare* females carrying the *f* element.

In conclusion, we identified a set of 35 *Wolbachia w*VulC genes that may be involved in the induction of cytoplasmic sex determination in the terrestrial isopod *A. vulgare*. Our results open the avenue for further functional analyses of these candidate genes, which may provide additional insights into the molecular genetic basis and cellular mechanisms of feminization. Of the 35 genes, 2 genes (*w*Vul_1408 and *w*Vul_1821) presented an expression profile in the heterologous host *C. convexus* which is correlated with that in the natural host *A. vulgare* and showing a one-stage shift corresponding to the one-stage shift existing in the sexual development sequence of *A. vulgare* and *C. convexus*. While *w*Vul_1821 contains no known functional domain, *w*Vul_1408 contains two known functional domains, including an Ankyrin Repeat Domain and a protease domain. Although *w*Vul_1408 is absent from the *f* element, it nevertheless constitutes a prime candidate for being involved in *Wolbachia w*VulC-mediated feminization. While *w*Vul_1408 may constitute a top candidate in subsequent studies aiming at unraveling the molecular genetic basis of feminization, it may also be important to invest efforts in the identification of the function of other candidate genes such as *w*Vul_1821. This will ultimately allow us to better understand the impact of *Wolbachia* endosymbionts on the mechanisms of sex determination of their terrestrial isopod hosts. It will also shed new light on the role of *Wolbachia* in sex chromosome turnovers experienced by *A. vulgare* in particular [16], and terrestrial isopods in general [94].

## Figures and Tables

**Table 1:** *Wolbachia* genome features with *w*Con and *w*VulC.

Supergroup	B	B	B	B	A	A	A	C	D	F
Strain	*w*Con	*w*VulC	*w*No	*w*Pip-Pel	*w*Mel	*w*Ri	*w*Ha	*w*Oo	*w*Bm	*w*Cle
Host	*Cylisticus convexus*	*Armadillidium vulgare*	*Drosophila simulans*	*Culex quinque-fasciatus*	*Drosophila melanogaster*	*Drosophila simulans*	*Drosophila simulans*	*Onchocerca ochengi*	*Brugia malayi*	*Cimex lectularius*
**Size (bp)**	2,109,518	1,663,741	1,301,823	1,482,455	1,267,782	1,445,873	1,295,804	957,990	1,080,084	1,250,060
**G + C%**	34.69	34.49	34.50	34.60	35.46	35.40	35.34	32.10	35.20	36.30
**Coding sequences**	2356	1888	1040	1275	1195	1150	1010	842	805	1216
**Coding base density**	0.78	0.83	0.80	0.86	0.94	0.80	0.78	0.67	0.75	0.75
**Gene average size**	702	725	1013	951	851	976	1000	757	899	771
**rRNA**	3	3	3	3	3	3	3	3	3	3
**tRNA**	35	35	34	34	34	34	34	34	34	34
**Pseudogenes**	NA	375	95	110	74	114	93	196	98	109

**Table 2:** Functional domains found in the 35 candidate genes. Underlined genes are present in the *f* element.

Gene Number	Annotation	Brief Description
67; 85; 375	Smc	Chromosome Segregation
375	AAA	DNA breaks
375	Spc7	Chromosome Segregation
67	Hook	N-term binding to microtubules
85	Mitofilin	Mitochondria inner membrane protein
359	OMPA-like	Surface
1360	IncA	Recruitment of lipids
1408; 1775	Trp	Transient receptor channels
493	LRR	Eukaryotic-like domain
881	TPR	Eukaryotic-like domain
303; 1408; 1775	ANK	Eukaryotic-like domain
873	Lipase	Hydrolizetriglycerides
1408; 1454; 1775	Peptidase/protease	Hydrolise peptide bonds
1775	p53	Bind to DNA, eukaryotic-like domain

## Supplementary Materials

The following are available online at http://www.mdpi.com/2073-4425/9/6/290/s1. Figure S1: Circular DNA plot of the *w*VulC genome; Figure S2: Distribution of COG families; Figure S3: Heatmap of the logratio of 139 *w*VulC gene expression during *A. vulgare* developmental stages; Figure S4: Cross-correlation values of 35 candidate genes from *Wolbachia w*VulC; Table S1: Primers and PCR conditions; Table S2: Primers and PCR conditions of candidate genes; Table S3: Categorization of 1888 *w*VulC genes; Table S4: Functional annotation of 35 candidate genes; Table S5: BLAST output of *w*VulC candidate genes in the *f* element.
