# Supplementary material for: Investigating the Molecular Genetic Basis of Cytoplasmic Sex Determination Caused by Wolbachia Endosymbionts in Terrestrial Isopods
Source: Genes (Basel). 2018 Jun 8;9(6):290. doi: 10.3390/genes9060290 (PMC6026926; doi:10.3390/genes9060290)
Supplement: Supplementary file 1 [file genes-09-00290-s001.zip › genes-292060-supplementary-legend-resubmission.docx]

Legends to Supplemental Figures

Figure S1: Circular DNA plot of the *w*VulC genome with position and categorization of each gene. From the exterior to the inner circle, the first colored track is for *in silico* classification, the second track for genes with no amplification or expression, the third track for regularly expressed genes in *Armadillidium vulgare* (AV) and the fourth track is for the 35 candidate genes, including all genes that are expressed or not in the *Cylisticus convexus* (CC) host.

Figure S2: Distribution of COG Families (Categorical Orthological Genes) in 7 *Wolbachia* genomes (*w*Con, *w*Ha, *w*Mel, *w*No, wPipPel, *w*Ri and *w*VulC). COGs were attributed to protein sequences by Blastp. COG Families are classified as follows: (i) Information, Storage and Processing ([J] Translation, ribosomal structure and biogenesis, [A] RNA processing and modification, [K] Transcription, [L] Replication, recombination and repair, and [B] Chromatin structure and dynamics), (ii) Cellular Processes and Signaling ([D] Cell cycle control, cell division, chromosome partitioning, [Y] Nuclear structure, [V] Defense mechanisms, [T] Signal transduction mechanisms, [M] Cell wall/membrane/envelope biogenesis, [N] Cell motility, [Z] Cytoskeleton, [W] Extracellular structures, [U] Intracellular trafficking, secretion, and vesicular transport, and [O] Posttranslational modification, protein turnover, chaperones), (iii) Metabolism ([C] Energy production and conversion, [G] Carbohydrate transport and metabolism, [E] Amino acid transport and metabolism, [F] Nucleotide transport and metabolism, [H] Coenzyme transport and metabolism, [I] Lipid transport and metabolism, [P] Inorganic ion transport and metabolism, and [Q] Secondary metabolites biosynthesis, transport and catabolism) and (iv) Poorly Characterized ([R] General function prediction only, [S] Function unknown and [none] those without any hit.)

Figure S3: Heatmap of the logratio of 139 *w*VulC gene expression during *A. vulgare* developmental stages 3 to 6 and calibrated on stage 2. The dendogram was obtained with p-distances using the gplots library of the R software.

Figure S4: Cross-correlation values of 35 candidate genes from *Wolbachia w*VulC which are differentially expressed in the isopod hosts *Armadillidium vulgare* and *Cylisticus convexus*. The Shift between *C. convexus* and *A. vulgare* developmental stages is on the x-axis (e.g. a shift of -1 means that for the *A. vulgare* stage 2 corresponds to *C. convexus* stage 1). The result of the cross-correlation function (CCF) is on the y-axis. Dotted blue lines represent the thresholds above which the correlation is significant (taken as 95% of the critical value of the distribution law [1.96] divided by the square root of the number of time points [7]). The threshold is equal to 0.74 and corresponds to *p*=0.05. Four *w*VulC genes overcrossed this threshold with positive correlation between the 2 hosts: wVul_0067, wVul_0881, wVul_1408 and wVul_1821. Next, to account for the sampling variance that was lacking in the cross-correlation function, the correlation ratio η² was computed for these 4 genes for both hosts (η² values are indicated in the frames of the relevant genes). If the value is >0.9, then the correlation is not significant.
