# Supplementary figures and images for: Investigating the Molecular Genetic Basis of Cytoplasmic Sex Determination Caused by Wolbachia Endosymbionts in Terrestrial Isopods
Source: Genes (Basel). 2018 Jun 8;9(6):290. doi: 10.3390/genes9060290 (PMC6026926; doi:10.3390/genes9060290)

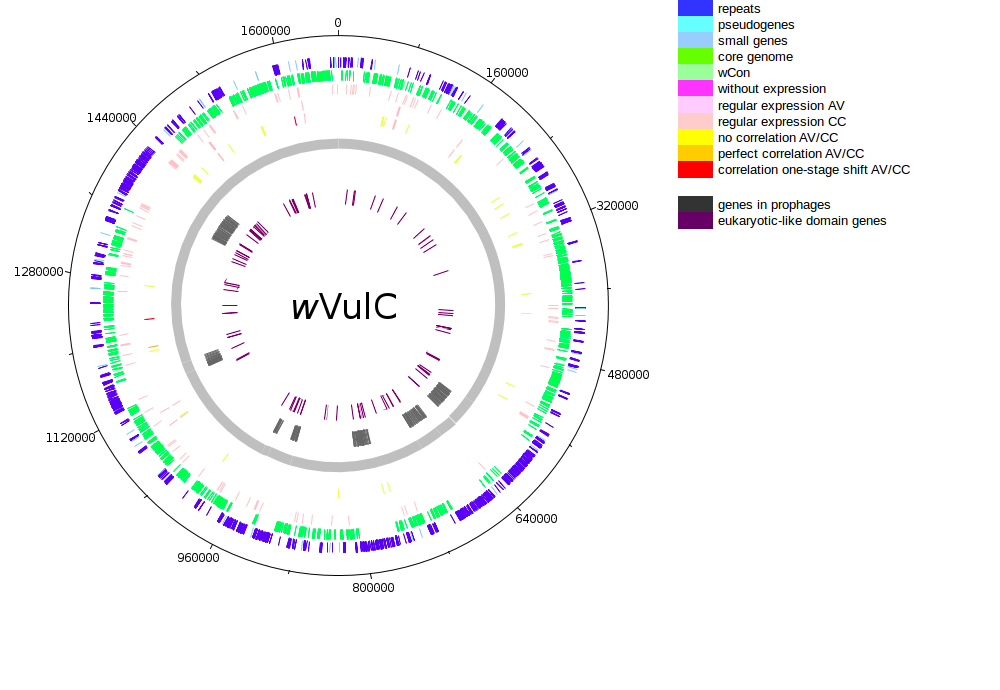

Supplement: Supplementary file 1 [file genes-09-00290-s001.zip › FigureS1.tif]

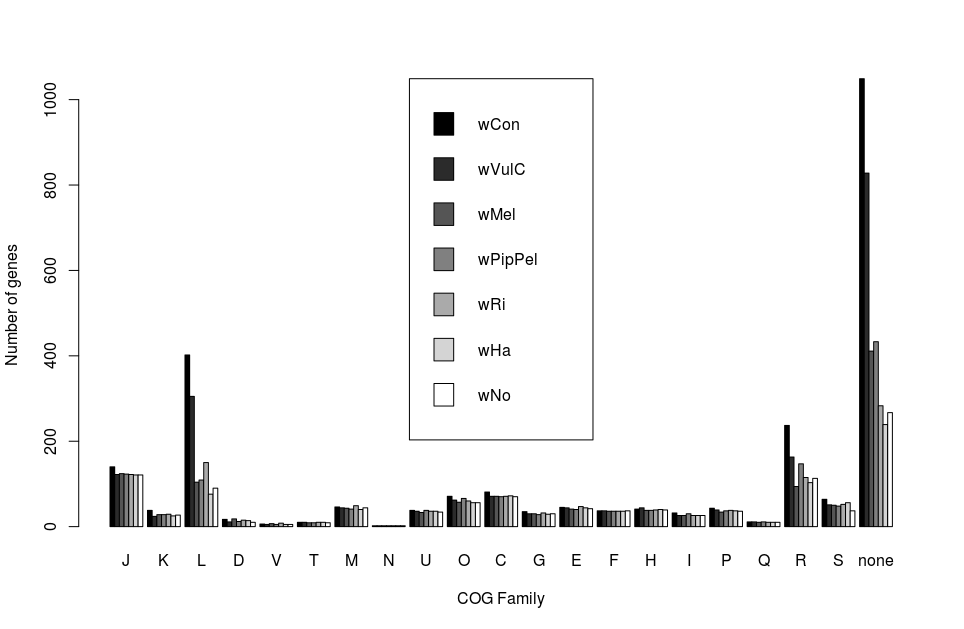

Supplement: Supplementary file 1 [file genes-09-00290-s001.zip › FigureS2.tiff]

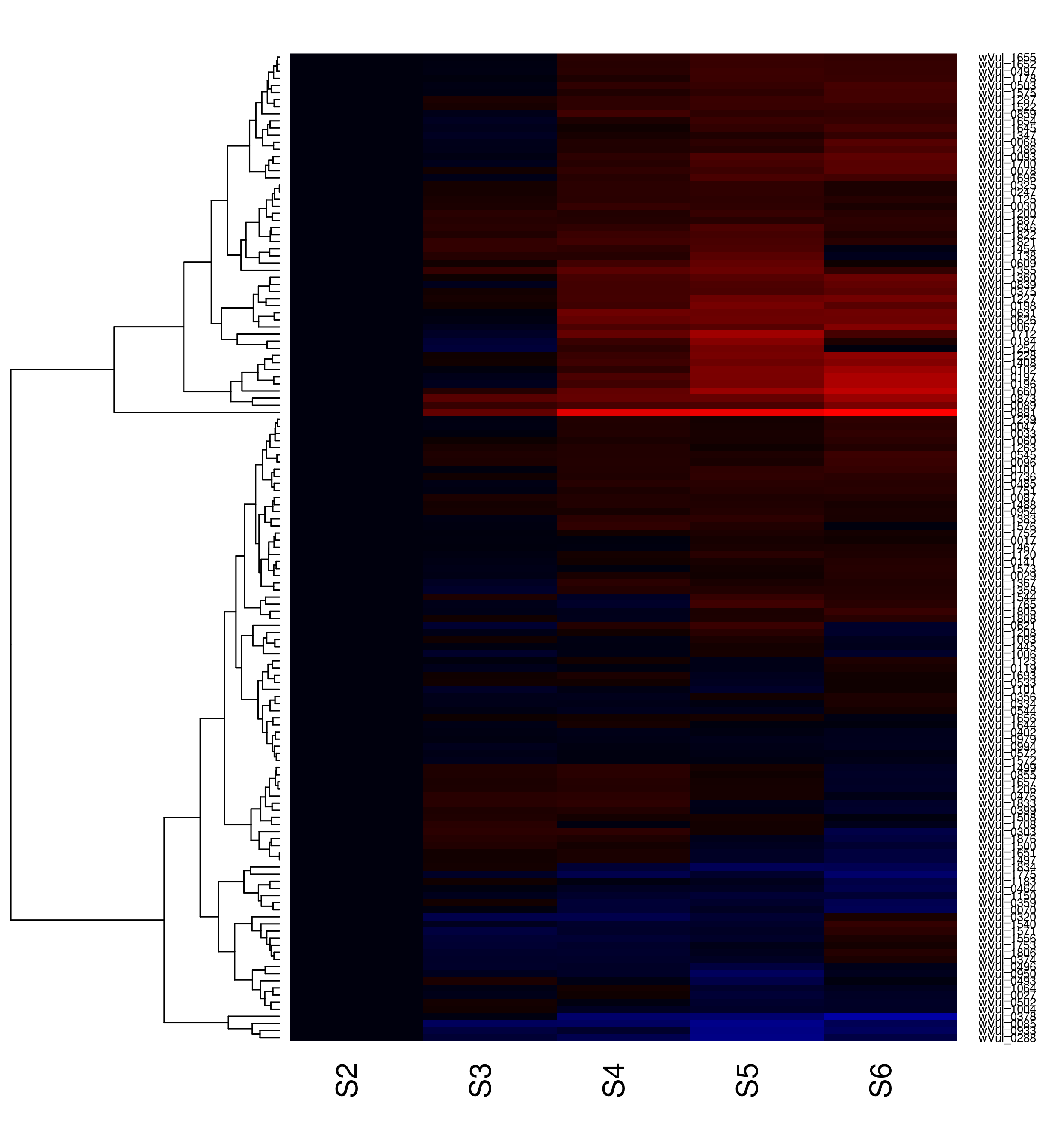

Supplement: Supplementary file 1 [file genes-09-00290-s001.zip › FigureS3.tiff]
